# Maternal status regulates cortical responses to the body odor of newborns

**DOI:** 10.3389/fpsyg.2013.00597

**Published:** 2013-09-05

**Authors:** Johan N. Lundström, Annegret Mathe, Benoist Schaal, Johannes Frasnelli, Katharina Nitzsche, Johannes Gerber, Thomas Hummel

**Affiliations:** ^1^Monell Chemical Senses CenterPhiladelphia, PA, USA; ^2^Department of Clinical Neuroscience, Karolinska InstitutetStockholm, Sweden; ^3^Department of Psychology, University of PennsylvaniaPA, USA; ^4^Department of Otorhinolaryngology, Smell and Taste Clinic, Technical University of DresdenDresden, Germany; ^5^Developmental Ethology and Cognitive Psychology Group, Centre des Sciences du Goût (Unité Mixte de Recherche 6265), Centre National de la Recherche ScientifiqueDijon, France; ^6^CERNEC, Département de Psychologie, Université de MontréalMontréal, QC, Canada; ^7^Department of Obstretrics and Gynecology, Technical University of DresdenDresden, Germany; ^8^Department of Neuroradiology, Technical University of DresdenDresden, Germany

**Keywords:** body odor, bonding, fMRI, neonatal, reward

## Abstract

Studies in non-human mammals have identified olfactory signals as prime mediators of mother-infant bonding and they have been linked with maternal attitudes and behavior in our own species as well. However, although the neuronal network processing infant cues has been studied for visual and auditory signals; to date, no such information exists for chemosensory signals. We contrasted the cerebral activity underlying the processing of infant odor properties in 15 women newly given birth for the first time and 15 women not given birth while smelling the body odor of unfamiliar 2 day-old newborn infants. Maternal status-dependent activity was demonstrated in the thalamus when exposed to the body odor of a newly born infant. Subsequent regions of interest analyses indicated that dopaminergic neostriatal areas are active in maternal-dependent responses. Taken together, these data suggests that body odors from 2 day-old newborns elicit activation in reward-related cerebral areas in women, regardless of their maternal status. These tentative data suggests that certain body odors might act as a catalyst for bonding mechanisms and highlights the need for future research on odor-dependent mother-infant bonding using parametric designs controlling for biological saliency and general odor perception effects.

The natural body odor of humans consists of a wide range of volatile and non-volatile compounds (Zeng et al., 1996) that carry cues conveying such disparate information as individual, gender, age, or kin identity (Weisfeld et al., 2003; Lundstrom et al., 2009; Mitro et al., 2012), physiological, stress, and disease states (McCulloch et al., 2006), and may direct mate selection and parental investment (Lundstrom and Jones-Gotman, 2009). Indeed, most of the information that humans attain by visual and auditory means are available in chemical signals and, even for humans, these chemical signals may convey much more information and influence behavior in ways that are still not appreciated (Shepherd, 2011).

Body odors are commonly viewed as a negative and large amount of financial resources and efforts are daily dedicated to either hiding or eliminating them (Gilbert and Firestein, 2002). However, it is often forgotten that certain body odors can also be viewed as immensely positive where one of the more salient and pleasurable experiences reported is the body odor originating from a new born child (Schaal et al., 1980). To date, human mother's behavioral response to neonatal body odor is well-established and like other neonatal traits, odors indeed seem to be particularly salient stimuli to post-parturient women (Schaal et al., 1980). Reciprocally, infants are highly reactive to maternal odors (Doucet et al., 2009). Such facts support the notion that body odors serve as a medium for the mutual exchange of cues and signals that may influence mother to infant and infant to mother signaling in a manner previously demonstrated for visual stimuli (Alley, 1981). However, studies identifying the neural processing of sensory signals mediating mother-infant bonding have focused near exclusively on visual and auditory signals, meaning that very little is known of the neural processing of bonding cues conveyed via our other senses. Immediately postpartum, odor-based cues direct the newborn's orienting decisions in the environment afforded by the mother's body (Schaal et al., 2004). This process is bidirectional and human adult caretakers experience heightened bonding in response to infant sensory cues as well as to infant-elicited behavior. This in turn releases nurturing attitudes and responses, and the correlative neural and neuroendocrine cascades, mainly within the dopaminergic reward system (Insel and Young, 2001).

Animal studies have addressed the neural substrates underlying a mother's responses to her infant's body odor in non-human animals (for a detailed review, see: Krasnegor and Bridges, 1990); however, no such study is at hand for our own species. The main regulator of reward guided learning in humans is the dopaminergic system (Schultz et al., 1997) with areas within the neostriatum (caudate nucleus and putamen) seen as regulators of the gradual, incremental learning of rewarding associations (Knowlton et al., 1996). Here, we employed functional magnetic resonance imaging (fMRI) to measure mothers brain responses to infants' body odors in a first attempt to assess whether infants' body odors promotes infant-caretaker bonding, i.e., reward based bonding mechanism, akin to what has been reported in the animal literature.

Assessing perceptual reward outside animal models is an inherently difficult task that can only be done by indirect verbal or psychophysiological measures. Verbal assessment of the levels of reward associated with viewing or smelling an infant is difficult and naturally fraught with a societal expectation that bias the individual toward a more positive evaluation than experienced (Callan, 1985). Similarly, assessing reward associations using psychophysiological measures, such as fMRI, without a strong theoretical assumption of reward based processing is indirect at best and often based on inverse inference. Therefore, based on the assumption that women newly given birth for the first time (primiparous) would demonstrate a stronger reward-oriented response to the body odor of an infant than women not given birth (nulliparous), we assessed differences between primiparous and nulliparous women in their neural processing of body odors from newly born infants.

## Material and methods

### Participants

Thirty healthy right-handed, non-smoking women participated. Fifteen were nulliparous (age range: 19–26 years; mean ± *SD*: 22.1 ± 1.9 years) and 15 were primiparous, having given birth 3–6 weeks prior to scanning (age range: 23–36 years; mean: 28.6 ± 4.1 years). The participating primiparous women were older than the nulliparous women [independent Student's *t*-test, *t*_(28)_ 5.4, *p* < 0.01]. The post-parturient women were recruited during their stay in the maternity at the Department of Gynecology at University of Dresden Medical School. They had all undergone a healthy pregnancy and delivered vaginally without complication. At the time of fMRI scanning and testing for infants' odor, all of the mothers were breastfeeding. Absence of anosmia in both groups of women were determined using the “Sniffin' Sticks” screening set comprising a 12 items odor identification test (Hummel et al., 2001) and an ear-nose-throat (ENT) examination. Olfactory identification performance did not significantly differ between the two groups.

All subjects provided written informed consent for their participation prior to testing and all aspects of the study were approved by the Ethics Committee of the Medical Faculty of the Technical University of Dresden and performed in accord with the Declaration of Helsinki on Biomedical Studies Involving Human Subjects.

### Odor stimuli and delivery

Neonatal body odors were collected by means of 100% cotton undershirts from 18 newly born infants. The infants slept in the undershirt for the first two nights postpartum at the post-delivery ward. After being worn, undershirts were immediately placed in odorless zip-lock plastic bag and frozen at −80°C for a maximum of 6 weeks. Deep freezing prevented the percept of the sampled body odors from changing over time. All undershirts were previously washed with an odorless wash powder using standardized procedures before wearing (Lundstrom et al., 2008). One hour prior presentation to the participants, the undershirts were thawed and placed in exposure vessels, so-called “gas washing bottles” of 250 ml volume (NeoLab, Heidelberg, Germany).

All stimuli were presented birhinally using a custom-built olfactometer. The olfactometer design was based on the same general principle as a previously published air-dilution olfactometer (for extensive description, cf. Lundstrom et al., 2010) and used a constant flow of humidified, odorless air (3 l/min) which was delivered through Teflon tubing terminating in Teflon nose pieces with inner diameters of 4 mm.

### Imaging design, procedures, and fMRI parameters

The study was performed using a 1.5 Tesla MR-scanner (Sonata; Siemens, Erlangen, Germany). For anatomic overlays, a T1-weighted (turboflash sequence) axial scan with 224 slices, voxel size of 1.6 × 1.1 × 1.5 mm, a repetition time (TR) of 2130 ms, echo time (TE) of 3.93 ms was acquired. Acquisition of blood-oxygen-level dependent (BOLD) signal was performed in the axial plane (oriented parallel to the planum sphenoidale to minimize bone artifacts) using a multi-slice spin-echo echo-planar imaging (SE-EPI) sequence. Scan parameters included a 64 × 64 matrix, voxel size of 3 × 3 × 3.75 mm, TR of 2630 ms, and a TE of 45 ms using a total of 24 slices.

**Figure 1:**
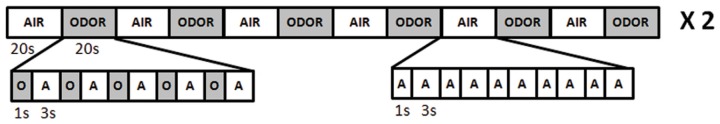
**Schematic overview of the olfactory presentation protocol for the imaging session**.

The scanning consisted of two identical runs (Figure 1). Within each run, participants were exposed to 6 body odor blocks, each 20 s long, and 6 odorless air blocks, also 20 s of length. Within each block, the stimulus (either body odor or odorless air) were delivered for 1 s every 4 s with odorless air in-between. This intermittent on-off paradigm was employed to reduce potential adaption and habituation. In reality, in the “odorless air” blocks, this meant that stimuli shifted between odorless air and odorless air but which acted as a control for potential tactile activation due to the weak alteration in airflow when shifting between stimuli. Each participant received the body odor originating from two different newborns in a randomized order. Each run contained both body odors and the primiparous women were not stimulated with body odor originating from their own infant to prevent a difference in identification between the two subject groups. At the very end of the experiment, one additional run consisting of stimuli unrelated to body odor processing were collected (not presented here). Odors were presented without a cue and participants were asked to breathe solely through their mouth by performing the velopharyngeal closing breathing technique (Kobal, 1981), a technique that removes sniff related effects and has been used extensively in previous fMRI and EEG odor studies (Lundstrom et al., 2006; Frasnelli et al., 2012). Although it is impossible to completely eliminate the potential influence of the cognitive awareness of the odor's identity, subjects were never informed of the stimulus identity. Following presentation of each run, subjects rated the average intensity, familiarity, and pleasantness of the stimuli on an 11-point category scales (Intensity and Familiarity: 10 = very intense/very familiar; 0 = odorless/very unfamiliar; Pleasantness: +5 = very pleasant; 0 = neutral; −5 = very unpleasant). In subsequent analyses, the pleasantness scale was converted into a positive scale with −5 being indicated by 0 and +5 by 10.

### Analyses and data reduction

Neuroimaging data were pre- and post-processed using SPM5 (Wellcome Department of Cognitive Neurology, London, UK, implemented in Matlab 7.1; MathWorks, Inc., Natick, MA, USA). Functional data were realigned, motion corrected, re-sliced, and coregistered to the individual T1 volume by means of segmentation fitting. Analyses were done on spatially normalized (stereotactically transformed into MNI ICBM152-space) and smoothed images (8 mm full width at half maximum (FWHM) Gaussian kernel) with a final voxel size of 3 × 3 × 3 mm. In this first level analysis, condition-specific beta values (parameter estimates) were estimated for both the body odor condition and odorless air condition on an individual level by entering the two runs as separate sessions and using the full 20 s blocks as event of interest for each condition. We then contrasted body odor condition vs. odorless air by the using the estimated movement parameters, obtained from the motion correction step described above, as regressors of no interest and filtered with high-pass filter (cut-off of 128 s).

At the group level, we assessed potential cerebral differences between primiparous and nulliparous in processing of newborns body odor, including age as a variable of no interest in all analyses to remove age-differences, in three ways. We first assessed differences between the two groups using a between-group t-contrast for the body odor activity obtain within the first-level analyses. We then assessed significant activation for the whole group using a simple t-contrast for the first level contrast body odor vs. odorless air. Based on our a priori hypothesis, we employed small volume corrections (SVC) for areas within neostriate cortex surviving an uncorrected threshold of *p* < 0.001. We subsequently performed directed region of interest analyses within areas of significance in the whole group analyses by extracting parameter (beta) values at the specific neostriate areas of interest using an 8 mm search sphere. We then assessed differences within these areas, based on the extracted parameter estimates, using paired-samples *t*-tests and Pearson correlations in the statistical software SPSS. Finally, as a control for general group differences, we assessed whether the two groups differed in respect of their neural response to the clean air condition within a separate model using a simple t-contrast to maximize power. All imaging statistical analyses were thresholded using a cluster criterion of three voxels and whole brain analyses corrected using a false discovery rate (FDR) of *p* < 0.05 unless otherwise noted.

## Results

**Figure 2:**
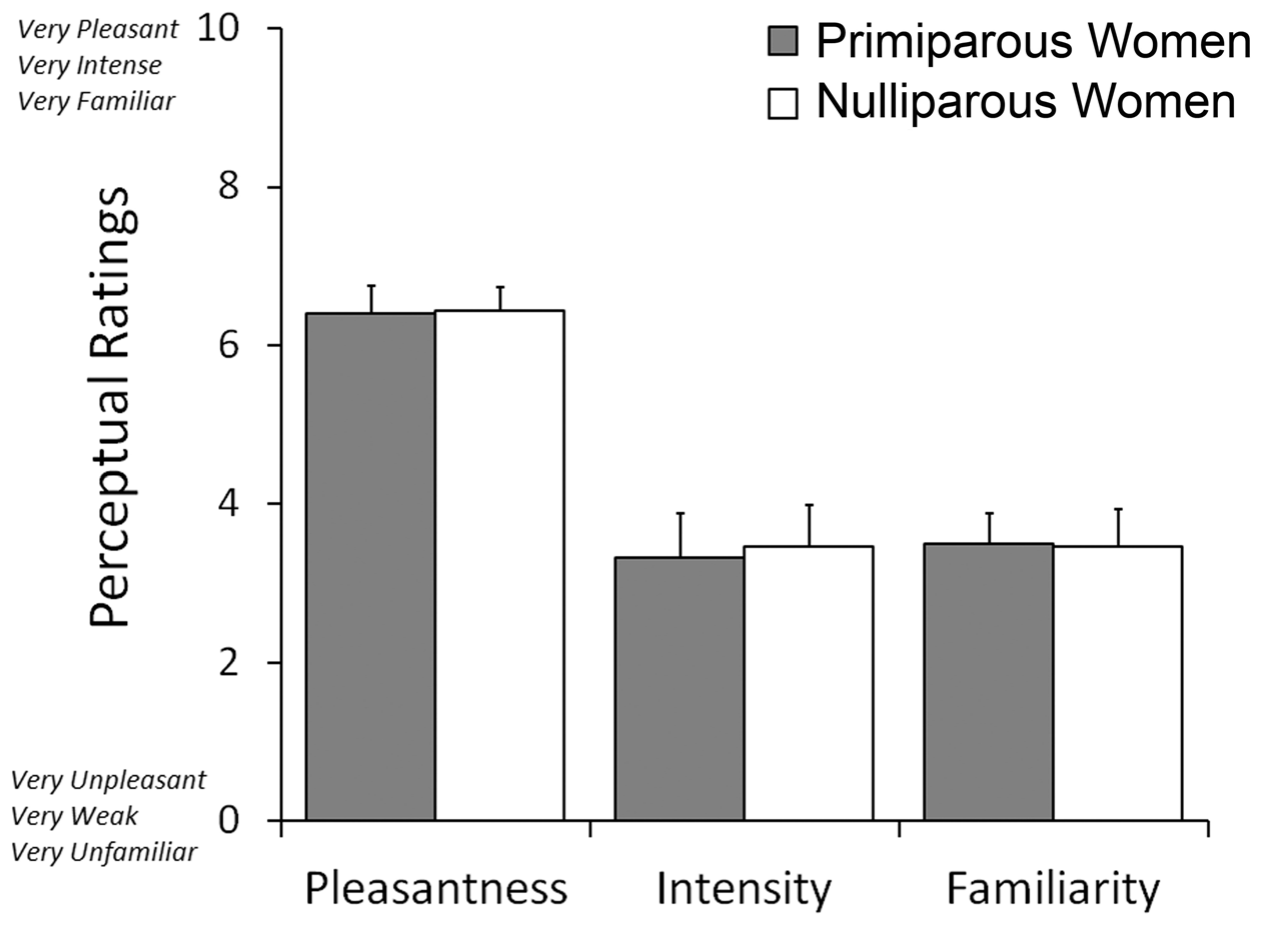
**Mean ratings of odor pleasantness, intensity, and familiarity judgments of post-parturient primiparous mothers and nulliparous women toward the body odors of 2-day old unfamiliar neonates**.

The participating women rated the neonatal body odors as weak, unfamiliar, and mildly pleasant. On a scale where low values indicate weak, unpleasant, and unfamiliar ratings, the body odor was rated as 3.4 ± 0.37 (mean ± SEM) for intensity, 6.4 ± 0.23 for pleasantness, and 3.4 ± 0.29 for familiarity (Figure 2). There was no significant difference between the two body odor presentations runs for any of the perceptual ratings as deemed by separate paired Student's *t*-tests. Moreover, there was no significant difference between primiparous and nulliparous women in how intense (3.3 vs. 3.4), how pleasant (6.4 vs. 6.4), or how familiar (3.5 vs. 3.4) they perceived the body odors to be [all *t* < 0.17, all *p* > 0.86].

We initially explored potential differences between primiparous and nulliparous women in their processing of newborns body odor. Primiparous women expressed a significantly greater activation in the thalamus with no other activations withstanding statistical whole brain correction (Table 1). The reverse contrast (nulliparous vs. primiparous women) did not produce any activity withstanding whole-brain correction.

**Table 1:** **Peaks of increased BOLD activation separated by contrast**.

**Area**	**MNI coordinates**			***Z*-value**
	***x***	***y***	***z***	
**CONTRAST PRIMIPAROUS vs. NULLIPAROUS WOMEN**				
Thalamus	−3	−5	3	4.53
**CONTRAST INFANTS BODY ODOR vs. ODORLESS AIR**				
***Cerebral areas***				
Hippocampus	21	−42	0	4.44
Insular cortex	−36	−33	21	4.09
Lateral orbitofrontal cortex	39	18	−14	3.79^*^
***Striate areas***				
Putamen	32	2	12	4.01^*^
Ventral caudate nucleus	−13	3	12	3.77^*^
Dorsal caudate nucleus	−16	−4	16	3.64^*^

Results marked by

* indicate that result is based on small volume correction (SVC).

**Figure 3:**
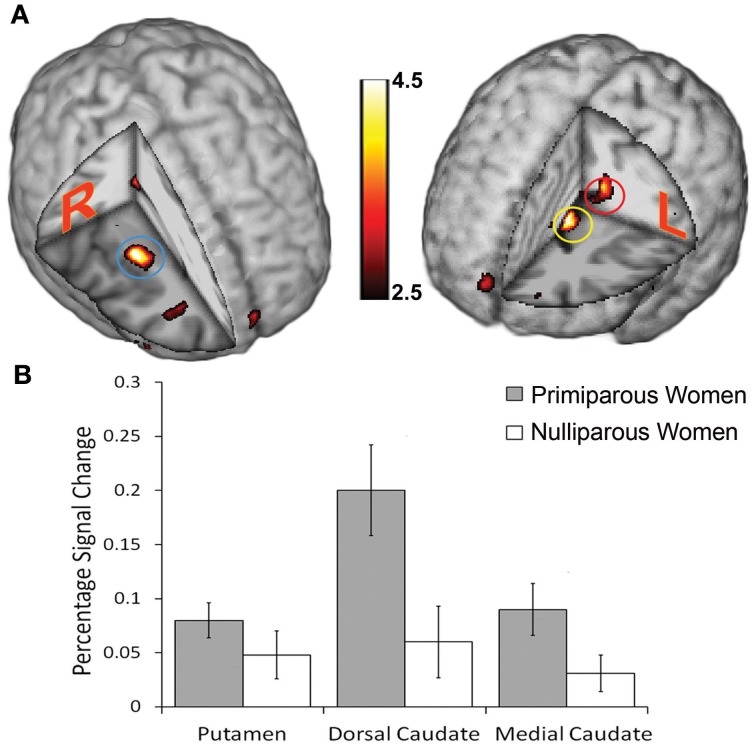
**Cerebral activations evoked in women smelling the body odor of an unfamiliar newborn**. **(A)** Blue circle marks the location of increased activation in the putamen; red circle marks increased activation in the dorsal caudate nucleus; yellow circle marks increased activation in the medial caudate nucleus. Display thresholded at *z* = 2.5, to demonstrate extent of activations, and activation superimposed on an anatomical template. Color scale indicates statistical *z*-values and absolute values can be found in Table 1. **(B)** Plots of percentage signal change for peak activity in the above locations for mothers and controls separately. Bars in graph represent standard error of the mean.

We then assessed cerebral responses to infants' body odor in all women. During the administration of the neonatal body odor, the participating women demonstrated an increase in neuronal response in the putamen, and the medial and dorsal caudate nucleus (Table 1; Figure 3A). To compare the two groups specifically within the areas of interest, we did directed region of interest analyses on the extracted beta-values. Separate Student's *t*-tests illustrated significant differences in both the medial [*t*_(28)_ = 2.06, *p* < 0.05] and dorsal [*t*_(28)_ = 2.57, *p* < 0.05] caudate nucleus, but not in the putamen (Figure 3B), between primiparous and nulliparous women. There were no significant correlations between perceptual ratings and activity with the neostriate areas [all *r* < 0.39, all *p* > 0.05]. Similarly, there was no area of significance between the primiparous and nulliparous women in respect of neural processing of the clean air condition even using a liberal uncorrected threshold of *p* < 0.001.

## Discussion

These data provides the first demonstration that neural processing of infants body odors are dependent on maternal status. Although exposed to stimuli of weak perceptual strength, the two groups differed in thalamic processing. These data also provides tentative support for our hypothesis that akin to other animals, cerebral reward learning networks are activated by the detection of an infant's body odor. The participating women, independent of maternal status, demonstrated increased processing in the neostriate areas, thus suggesting that a 2 day-old newborn infant's body odor may convey cues that can motivate affect in parent or non-parent females to care for unrelated and unfamiliar infant alike.

Although the participating women displayed a significant response to infants' body odors in neostriate areas, the lack of an odor control that exhibit an equal amount of biological reward as the infants body odor, such food or other ecological relevant odors, means that we cannot directly demonstrate that this is an effect attributable to infant body odor. However, the direct comparison between the two groups for identical odors does not suffer from this potential confounding factor. This comparison demonstrated a tentative dissociative parental status-dependent pattern within neostriate areas. Although the neostriate area is often referred to as one entity, clear and dissociable roles exist within. Whereas the putamen is often linked to implicit learning (Packard and Knowlton, 2002), the dorsal caudate nucleus has been tightly linked with stimulus-response link learning in both instrumental conditioning and reinforcement studies (O'Doherty et al., 2004). It is interesting to note that all participating mothers, individuals who had experienced more recent, longer, and more affectively-loaded exposure to neonatal body odor than the controls, demonstrated higher activity in the dorsal caudate nucleus whereas there was no difference in the putamen. This tentative dissociation in the dorsal caudate, with mothers expressing higher activation, suggests that mothers are more tuned to the reinforcement process that the interactions with an infant might lead to in comparison with nulliparous women. This in turn may lead to an enhanced reward learning mechanism as demonstrated in several other animals (Packard, 1999). The statistical difference between the two groups of women did not survive a so-called statistical whole brain correction for multiple statistical testing; the demonstrated differences are based on region of interest analyses. It is, however, prudent to point out that these additional analyses cannot be assumed to be circular, as defined by Kriegeskorte and colleagues (2009), since they are based on a priori defined subject grouping unrelated to the analyses in question. Whether these differences are due to a learned response or to other processes, such as a difference in perceived salience or attention to infant odors, and whether the similarity of responses in the putamen is mediated by a predisposed motivational brain mechanism, remains to be elucidated in future full scale studies. Thus, although these results indicate that areas commonly involved in reward processing were activated in response to body odors of a newborn infant, one should be aware that this is not a causal demonstration of a novel odor-mediated bonding mechanism. It is interesting to note, however, that this effect appears similar to the undifferentiated brain responses of adults toward babies' faces (Glocker et al., 2009). Future studies are needed to rule out other potential explanation of these results by experimentally manipulating the biological reward that these chemosignals might communicate.

The body odor of infants also activated the orbitofrontal and insular cortices (Table 1). Both areas are often reported in neuroimaging studies of olfaction (Zatorre et al., 1992; Seubert et al., 2012) and their exact role in the olfactory system is currently not clear. Direct anatomical projection exists between the lateral orbitofrontal cortex and caudate nucleus as well as the ventral putamen; the two later areas are not commonly activated by common odors as recently demonstrated by a recent comprehensive meta analyses of all published olfactory neuroimaging studies (Seubert et al., 2012). Similarly, strong connections can also be seen between the insular cortex and medial putamen and medial caudate nucleus (Ongur and Price, 2000). Whether this intimate anatomical connection also implies a functional connection remains to be determined. It is interesting to note, however, that the insular cortex has repeatedly been involved in studies exploring cerebral processing of human body odors (Lundstrom et al., 2008, 2009; Prehn-Kristensen et al., 2009) as well as in a study exploring cerebral reward activation from viewing pictures of babies (Glocker et al., 2009). Whether this is an indication of a more general processing of body odors or whether it is an indication of other, related processes, such as emotional signals (Zhou and Chen, 2009), or just basic odor processing (Seubert et al., 2012) are ripe for future work.

We did not find any significant activation in the primary olfactory cortex (piriform cortex) when we contrasted body odors vs. air (although activation in the lateral orbitofrontal cortex was observed). One should note, however, that studies investigating the cerebral processing of body-related odorants repeatedly report a lack of activations in olfactory related cortices (Lundstrom et al., 2009; Mujica-Parodi et al., 2009; Prehn-Kristensen et al., 2009). Whether this means that body odors are partly or exclusively processed outside what is considered primary and secondary olfactory cortices remains to be elucidated.

In conclusion, the scent of a newborn infant is able to elicit increased responses in the brain's neostriatal areas within women that in previous studies have been closely linked with reward learning mechanisms (Kelley and Berridge, 2002). These findings tentatively suggest a potential reward mechanism by which bonding serves to elicit maternal motivational and emotional responses. A direct and strong causal link between biological reward and the findings presented in this experiment remains to be demonstrated in future experiments that directly and experimentally varying the degree of biological reward by means of food odors or other ecologically salient odors. These findings add to a growing literature that suggests that cues embedded within the complex mixture of body odors may be responsible for eliciting and/or supporting psychobiological processes.

### Conflict of interest statement

The authors declare that the research was conducted in the absence of any commercial or financial relationships that could be construed as a potential conflict of interest.

## References

[B1] Alley T. R. (1981). Head shape and the perception of cuteness. Dev. Psychol. 17, 650–654 10.1037/0012-1649.17.5.6509226933

[B2] Callan V. J. (1985). Perceptions of parents, the voluntarily and involuntarily childless: a multidimensional scaling analysis. J. Marriage Fam. 47, 1045–1050 10.2307/352349

[B3] Doucet S. Soussignan R. Sagot P. Schaal B. (2009). The secretion of areolar (Montgomery's) glands from lactating women elicits selective, unconditional responses in neonates. PLoS ONE 4:e7579 10.1371/journal.pone.0007579 19851461 PMC2761488

[B4] Frasnelli J. Lundstrom J. N. Schopf V. Negoias S. Hummel T. Lepore F. (2012). Dual processing streams in chemosensory perception. Front. Hum. Neurosci. 6:288 10.3389/fnhum.2012.00288 23091456 PMC3476497

[B5] Gilbert A. N. Firestein S. (2002). Dollars and scents: commercial opportunities in olfaction and taste. Nat. Neurosci. 5 Suppl., 1043–1045 10.1038/nn937 12403982

[B6] Glocker M. L. Langleben D. D. Ruparel K. Loughead J. W. Valdez J. N. Griffin M. D. (2009). Baby schema modulates the brain reward system in nulliparous women. Proc. Natl. Acad. Sci. U.S.A. 106, 9115–9119 10.1073/pnas.0811620106 19451625 PMC2690007

[B7] Hummel T. Konnerth C. G. Rosenheim K. Kobal G. (2001). Screening of olfactory function with a four-minute odor identification test: reliability, normative data, and investigations in patients with olfactory loss. Ann. Otol. Rhinol. Laryngol. 110, 976–981 11642433 10.1177/000348940111001015

[B8] Insel T. R. Young L. J. (2001). The neurobiology of attachment. Nat. Rev. Neurosci. 2, 129–136 10.1038/35053579 11252992

[B9] Kelley A. E. Berridge K. C. (2002). The neuroscience of natural rewards: relevance to addictive drugs. J. Neurosci. 22, 3306–3311 11978804 10.1523/JNEUROSCI.22-09-03306.2002 PMC6758373

[B10] Knowlton B. J. Mangels J. A. Squire L. R. (1996). A neostriatal habit learning system in humans. Science 273, 1399–1402 10.1126/science.273.5280.1399 8703077

[B11] Kobal G. (1981). Electrophysiologische Untersuchungen des menschlichen Geruchssinns. Stuttgart: Thieme Verlag

[B12] Krasnegor N. A. Bridges R. S. (1990). Mammalian Parenting. New York, NY: Oxford University Press

[B13] Kriegeskorte N. Simmons W. K. Bellgowan P. S. F. Baker C. I. (2009). Circular analysis in systems neuroscience: the dangers of double dipping. Nat. Neurosci. 12, 535–540 10.1038/nn.2303 19396166 PMC2841687

[B14] Lundstrom J. N. Boyle J. A. Zatorre R. J. Jones-Gotman M. (2008). Functional neuronal processing of body odors differ from that of similar common odors. Cereb. Cortex 18, 1466–1474 10.1093/cercor/bhm178 17934190

[B15] Lundstrom J. N. Boyle J. A. Zatorre R. J. Jones-Gotman M. (2009). The neuronal substrates of human olfactory based kin recognition. Hum. Brain Mapp. 30, 2571–2580 10.1002/hbm.20686 19067327 PMC6870682

[B16] Lundstrom J. N. Gordon A. R. Alden E. C. Boesveldt S. Albrecht J. (2010). Methods for building an inexpensive computer-controlled olfactometer for temporally-precise experiments. Int. J. Psychophysiol. 78, 179–189 10.1016/j.ijpsycho.2010.07.007 20688109 PMC2967213

[B17] Lundstrom J. N. Jones-Gotman M. (2009). Romantic love modulates women's identification of men's body odors. Horm. Behav. 55, 280–284 10.1016/j.yhbeh.2008.11.009 19118557

[B18] Lundstrom J. N. Olsson M. J. Schaal B. Hummel T. (2006). A putative social chemosignal elicits faster cortical responses than perceptually similar odorants. Neuroimage 30, 1340–1346 10.1016/j.neuroimage.2005.10.040 16413793

[B19] McCulloch M. Jezierski T. Broffman M. Hubbard A. Turner K. Janecki T. (2006). Diagnostic accuracy of canine scent detection in early- and late-stage lung and breast cancers. Integr. Cancer Ther. 5, 30–39 10.1177/1534735405285096 16484712

[B20] Mitro S. Gordon A. R. Olsson M. J. Lundstrom J. N. (2012). The smell of age: perception and discrimination of body odors of different ages. PLoS ONE 7:e38110 10.1371/journal.pone.0038110 22666457 PMC3364187

[B21] Mujica-Parodi L. R. Strey H. H. Frederick B. Savoy R. Cox D. Botanov Y. (2009). Chemosensory cues to conspecific emotional stress activate amygdala in humans. PLoS ONE 4:e6415 10.1371/journal.pone.0006415 19641623 PMC2713432

[B22] O'Doherty J. Dayan P. Schultz J. Deichmann R. Friston K. Dolan R. J. (2004). Dissociable roles of ventral and dorsal striatum in instrumental conditioning. Science 304, 452–454 10.1126/science.1094285 15087550

[B23] Ongur D. Price J. L. (2000). The organization of networks within the orbital and medial prefrontal cortex of rats, monkeys and humans. Cereb. Cortex 10, 206–219 10.1093/cercor/10.3.206 10731217

[B24] Packard M. G. (1999). Glutamate infused posttraining into the hippocampus or caudate-putamen differentially strengthens place and response learning. Proc. Natl. Acad. Sci. U.S.A. 96, 12881–12886 10.1073/pnas.96.22.12881 10536017 PMC23146

[B25] Packard M. G. Knowlton B. J. (2002). Learning and memory functions of the basal ganglia. [Review]. Annu. Rev. Neurosci. 25, 563–593 10.1146/annurev.neuro.25.112701.142937 12052921

[B26] Prehn-Kristensen A. Wiesner C. Bergmann T. O. Wolff S. Jansen O. Mehdorn H. M. (2009). Induction of empathy by the smell of anxiety. PLoS ONE 4:e5987 10.1371/journal.pone.0005987 19551135 PMC2695008

[B27] Schaal B. Hummel T. Soussignan R. (2004). Olfaction in the fetal and premature infant: functional status and clinical implications. Clin. Perinatol. 31, 261–285 15289032 10.1016/j.clp.2004.04.003

[B28] Schaal B. Montagner H. Hertling E. Bolzoni D. Moyse A. Quichon R. (1980). Olfactory stimulation in the relationship between child and mother. Reprod. Nutr. Dev. 20, 843–858 10.1051/rnd:19800510 7349450

[B29] Schultz W. Dayan P. Montague P. R. (1997). A neural substrate of prediction and reward. [Article]. Science 275, 1593–1599 10.1126/science.275.5306.1593 9054347

[B30] Seubert J. Freiherr J. Djordjevic J. Lundstrom J. N. (2012). Statistical localization of human olfactory cortex. Neuroimage 66C, 333–342 23103688 10.1016/j.neuroimage.2012.10.030

[B31] Shepherd G. M. (2011). Neurogastronomy: How the Brain Creates Flavor and Why it Matters. New York, NY: Columbia University Press

[B32] Weisfeld G. E. Czilli T. Phillips K. A. Gall J. A. Lichtman C. M. (2003). Possible olfaction-based mechanisms in human kin recognition and inbreeding avoidance. J. Exp. Child Psychol. 85, 279–295 10.1016/S0022-0965(03)00061-4 12810039

[B33] Zatorre R. J. Jones-Gotman M. Evans A. C. Meyer E. (1992). Functional localization and lateralization of human olfactory cortex. Nature 360, 339–340 10.1038/360339a0 1448149

[B34] Zeng X.-N. Leyden J. J. Spielman A. I. Preti G. (1996). Analysis of the characteristic human female axillary odors: qualitative comparison to males. J. Chem. Ecol. 22, 237–257 10.1007/BF02055096 24227407

[B35] Zhou W. Chen D. (2009). Sociochemosensory and emotional functions: behavioral evidence for shared mechanisms. [Article]. Psychol. Sci. 20, 1118–1124 10.1111/j.1467-9280.2009.02413.x 19686296 PMC2901506

